# The temporal dynamics of plasma fractalkine levels in ischemic stroke: association with clinical severity and outcome

**DOI:** 10.1186/1742-2094-11-74

**Published:** 2014-04-10

**Authors:** Gerrit M Grosse, Anita B Tryc, Meike Dirks, Ramona Schuppner, Henning Pflugrad, Ralf Lichtinghagen, Karin Weissenborn, Hans Worthmann

**Affiliations:** 1 Department of Neurology, Hannover Medical School, Carl-Neuberg-Str. 1, 30625 Hannover, Germany; 2 Department of Clinical Chemistry, Hannover Medical School, Carl-Neuberg-Str. 1, 30625 Hannover, Germany; 3 Center for Systems Neuroscience (ZSN), Bünteweg 2, 30559 Hannover, Germany

**Keywords:** Biomarker, CX3CL1, Fractalkine, Inflammation, Ischemic stroke, Outcome, Severity

## Abstract

**Background:**

The chemokine fractalkine (CX3CL1, FKN) is involved in neural-microglial interactions and is regarded as neuroprotective according to several* in vivo *studies of inflammatory and degenerative states of the brain. Recently, an association with outcome in human ischemic stroke has been proposed. In this study, we aimed to investigate the temporal pattern of FKN levels in acute ischemic stroke in relation to stroke severity and outcome.

**Methods:**

FKN levels were measured in plasma specimens of fifty-five patients with acute ischemic stroke. Blood was available for time points 6 hours (h), 12 h, 3 days (d), 7 d and 90 d after stroke onset. Clinical outcome was evaluated using the modified Rankin Scale (mRS) at 7 d and 90 d.

**Results:**

The time course of FKN significantly differs depending on stroke severity, with higher FKN levels linked to a lower severity. FKN levels in patients with moderate to severe strokes differ significantly from controls. In outcome analysis, we found an association of dynamics of FKN with clinical outcome. Decrease of FKN is pronounced in patients with worse outcome. Multivariate analysis including stroke severity and stroke etiology revealed that deltaFKN between 6 h and 3 d is independently associated with mRS at 90 d. In addition deltaFKN is inversely correlated with the extent of brain damage, as measured by S100B.

**Conclusions:**

FKN dynamics are independently associated with stroke outcome. Further studies might give insight on whether FKN is actively involved in the inflammatory cascade after acute ischemic stroke.

## Background

Fractalkine (CX3CL1, FKN) is the only chemokine with the CX3C motif and was first described in 1997 [1]. It appears to be unique because it exists in a membrane-bound, as well as in a soluble, form after being cleaved mainly through a disintegrin and metalloproteinase domain-containing protein 10 (ADAM10) [2]. FKN has chemotactic and adhesive functions for inflammatory cells. Its corresponding receptor, CX3CR1, is expressed mainly on monocytes, natural killer cells, T-lymphocytes and microglia.

FKN appears on the surface of endothelial cells and neurons and has been associated with several, mainly inflammatory, pathologies such as rheumatoid diseases [3], atherosclerosis, and neurodegenerative disorders like Alzheimer’s [4] and Parkinson’s disease [5]. In the brain, the CX3CL1/CX3CR1 axis takes part in neural-microglial signaling [6-8].

Recently, FKN has become notable in stroke research, primarily in animal models and in *in vitro* studies. The distinct roles of FKN and its receptor in cerebral ischemia are not yet sufficiently investigated and whether FKN adopts neuroprotective or neurotoxic functions remains controversial. Rodents treated with exogenous FKN after permanent occlusion of the middle cerebral artery (pMCAO) had smaller infarction sizes and less neurological deficits [9]. Controversially, genetically CX3CL1- and CX3CR1-deficient mice are also less susceptible to cerebral ischemia [9-11]. In inflammatory states of the brain, FKN probably acts via modulatory effects on microglia [12,13], which exert crucial activities in the response after ischemia. Thus, the interactions between neurons and microglia, mediated by the CX3CL1/CX3CR1 pathway could be essential in the pathology of stroke. In contrast to most other markers in stroke, FKN might, therefore, be less reactively driven by post-ischemic inflammation, but could be taking part in the process itself.

Donohue *et al*. recently proposed that higher FKN plasma levels are associated with better 180d-Outcome of ischemic stroke [14]. Based on these results we aimed to investigate the temporal pattern of FKN plasma levels in the hyperacute as well as long-term stage of ischemic stroke and whether FKN is independently associated with clinical outcome.

## Methods

### Study population

Fifty-five ischemic stroke patients were included into the current study. The patients derived from a cohort of a former study on inflammation markers in ischemic stroke that was successively recruited between January 2007 and April 2009 at the Department of Neurology at Hannover Medical School [15]. Patients with malignant tumor, immunological disease, cerebral hemorrhage and infections, as well as those with C-reactive protein (CRP) levels >50 mg/l, were excluded from further analysis.

Blood was drawn at time points: 6 hours (h), 12 h, 24 h, 3 days (d) and 7 d after stroke onset. For 50 patients, additional samples were available for 90 d after stroke onset.

In addition, measurements of fractalkine levels were performed in samples of 32 control subjects with cardiovascular risk factors (CVRF) but no history of cardiovascular events in the past 3 months. This was necessary to exclude a probable influence of CVRF in the comparison of patients with controls.

All specimens were centrifuged with 3,000 rpm for 10 minutes and then stored at -80°C until measurement.

The study was approved by the ethics committee of Hannover Medical School. All patients or legal representatives and volunteers provided written informed consent before inclusion in the study.

### Clinical evaluation

For every patient, demographic and clinical data such as cardiovascular risk factors (hypertension, diabetes mellitus, nicotine consumption and lipid dysregulation) or intravenous thrombolytic treatment with reverse tissue-type plasminogen activator (rtPA) were obtained. In addition, complete blood counts and creatinine levels were available for each stroke patient. For stroke severity, the National Institutes of Health Stroke Scale (NIHSS) has been obtained on admission. Patients were subdivided into two groups. According to the median, the cut-off was laid at NIHSS = 3. The groups were defined as mild (NIHSS ≤3) and moderate/severe (NIHSS >3) strokes [16]. The clinical outcome was categorized using the modified Rankin Scale at 7 d and 90 d after stroke onset. The outcome was defined as favorable (mRS: 0 to 1) or unfavorable (mRS: 2 to 6) [17,18]. The proportional differences of FKN levels (∆FKN) were calculated as the difference of the value at admission (6 h) and a later time point, divided by the 6-h value. Stroke etiology was categorized according to the results of Doppler ultrasound of the brain-supplying vessels, transthoracic/transesophageal echocardiography, and imaging studies of the brain (cranial computer tomography or magnetic resonance imaging) using the TOAST Criteria [19].

### Laboratory studies

FKN levels were determined in EDTA plasma using ELISA kits (R&D Systems, Inc., Minneapolis, MN, USA). The sensitivity of the assay was 0.018 ng/mL. Blood concentrations of the markers IL-6, S100B, CRP, MMP-9, TIMP-1 and MCP-1 had been measured in the course of previous studies of our group [15] and were available for controls and all patients at the same time points as mentioned above with few exceptions [for distinct patient numbers see Additional file 1: Table S3-8]. Measurements were made by researchers blinded to the patient’s characteristics.

### Statistical analysis

Statistical analysis was done using IBM SPSS Statistics 20 (SPSS Inc., Chicago, IL, USA) and GraphPad Prism 5 (GraphPad Software, Inc., La Jolla, CA, USA). Gaussian distribution was tested using the Kolmogorov-Smirnov test. Comparisons between groups were done using Student’s *t*-test for normally distributed data or Mann-Whitney *U*-test for non-normally distributed data and Chi-square test for categorical data. Correlations were calculated with bivariate Pearson correlation or Spearman correlation, as appropriate. Multivariate logistic regression was performed, including stroke severity and gender, as those were significantly different at baseline, and stroke etiology. This was done to exclude the probable influence of the underlying disease. A *P* value <0.05 was regarded as significant. For multiple testing, probability values were corrected using Bonferroni correction.

## Results

### Study population

The median age of the study population (n = 55) was 69 years and of the controls (n = 32) 71 years. For further clinical data and cardiovascular risk factors see Table 1. Gender differed between patients and controls (*P*<0.05). Other baseline characteristics did not differ.

**Table 1:** Baseline characteristics of the study population

**Parameters**	**Total stroke patients (n = 55)**	**Favorable outcome (n = 33)**	**Unfavorable outcome (n = 22)**	**Control (n = 32)**	***P *** **value**
**Gender**					**0.014**
Female	**25** (45%)	**10** (30%)	**15 **(68%)	**18 **(56%)	
Male	**30** (55%)	**23 **(79%)	**7 **(32%)	**14 **(44%)	
**Age (Years, Median) **(25th to 75th percentiles)	**69 **(62 to 77 )	**68 **(62 to 74)	**76,5 **(57,75 to 85)	**71 **(61,25 to 75,75)	**0.277**
**Etiology**					**0.258**
Large artery atherosclerosis	**8** (15%)	**3 **(9%)	**5 **(23%)	-	
Cardioembolism	**14 **(25%)	**7 **(21%)	**7 **(32%)	-	
Small vessel occlusion	**13 **(24%)	**10 **(30%)	**3 **(14%)	-	
Undetermined	**20 **(36%)	**13 **(39%)	**7 **(32%)	-	
**Stroke severity**					**0.001**
Mild	**28 **(51%)	**23 **(70%)	**5 **(23%)	-	
Moderate/severe	**27 **(49%)	**10 **(30%)	**17 **(77%)	-	
**SBP (mmHg, Median) **(25th to 75th percentiles)	**135 **(130 to 143)	**135 **(130 to 142)	**135 **(129 to 145,25)	**130 **(120 to 140)	**0.453**
**DBP (mmHg, Median) **(25th to 75th percentiles)	**80 **(70 to 86)	**80 **(70 to 88,5)	**80** (70 to 85)	**80 **(70 to 90)	**0.344**
**Hypertension**	**34** (62%)	**19 **(58%)	**15 **(68%)	**24 **(75%)	**0.325**
**Nicotine consumption**	**10 **(18%)	**7 **(21%)	**3 **(14%)	**6 **(19%)	**0.495**
**Dyslipoproteinemia**	**19 **(35%)	**12 **(36%)	**7 **(32%)	**17 **(53%)	**0.224**
**Diabetes mellitus**	**6 **(11%)	**3 **(9%)	**3 **(14%)	**4 **(13%)	**0.853**
**i.v. rtPA**	**8 **(15%)	**3 **(9%)	**5 **(23%)	**-**	**0.160**

Kruskal-Wallis test or Chi-square compared the groups (favorable, unfavorable strokes and controls). *P*<0.05 is considered significant. DBP, diastolic blood pressure; i.v. rtPA, intravenous reverse tissue-type plasminogen activator; SBP, systolic blood pressure,

### Levels of fractalkine in stroke patients compared to controls

**Figure 1:**
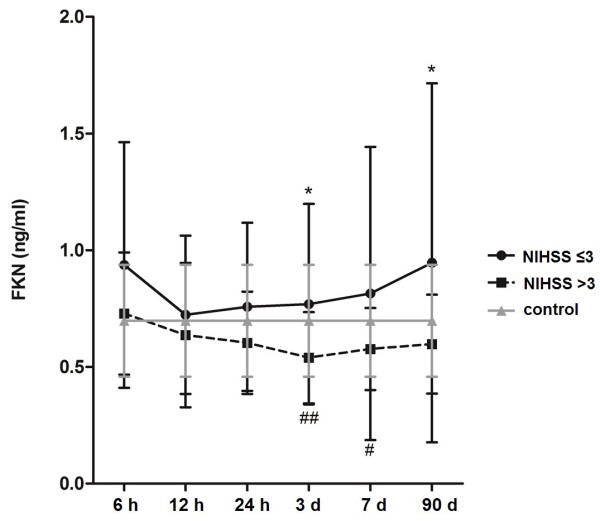
**Differences of fractalkine (FKN) levels at different time points after stroke onset depending on stroke severity (means ± SD). **Asterisks indicate significant differences between the two groups mild (NIHSS ≤3) and moderate/severe strokes (NIHSS >3). Number signs below the error bars indicate significant differences between moderate/severe stroke patients and controls. Within-group comparisons of FKN levels between the 6-hour (h) value and follow-up time points: significant differences were detected for the group of patients with mild strokes between 6 h and 12 h (*P*<0.001), 6 h and 24 h (*P* = 0.003), 6 h and 3 days (d) (*P*<0.001). For patients with moderate/severe strokes, significant differences were detected between 6 h and 24 h (*P* = 0.006), 6 h and 3 d (*P*<0.001), 6 h and 7 d (*P*<0.001) and 6 h and 90 d (*P* = 0.027).

Comparison of the values in stroke patients with those in controls with CVRF revealed the following differences: FKN levels did not differ from those in controls for the whole group of stroke patients. When patients with mild (NIHSS ≤3) and moderate/severe strokes (NIHSS >3) were analyzed separately, significant differences were observed. At time points 3 d (*P* = 0.008) and 7 d (*P* = 0.046), FKN levels of patients with moderate/severe stroke were significantly lower than those in controls and tended to be lower at 90 d (*P* = 0.082). On the contrary, levels in patients with mild stroke tended to be higher than in controls at 6 h (*P* = 0.055) (Figure 1).

### Time course of fractalkine depending on stroke severity

FKN levels differed at time points 3 d (*P* = 0.014) and 90 d (*P* = 0.025) and tended to vary at 7 d (*P* = 0.099) in patients with mild compared to moderate/severe stroke on admission (Figure 1). Regarding the temporal profile, FKN plasma levels decrease after ischemic stroke from the 6-h value until 7 d for the whole stroke patient group (*P*<0.001). For patients with moderate/severe stroke, the intragroup comparison showed that levels significantly decline, even until 90 d after stroke onset. Conversely, in patients with mild strokes, FKN reaches initial levels again after a shorter suppression of 3 d (Figure 1).

### Association of dynamics of fractalkine with stroke outcome

**Figure 2:**
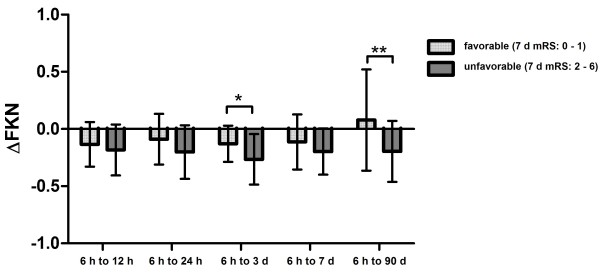
Proportional differences of fractalkine levels (∆FKN) at different time points after stroke depending on 7-day (d) stroke outcome (means ± SD).

**Figure 3:**
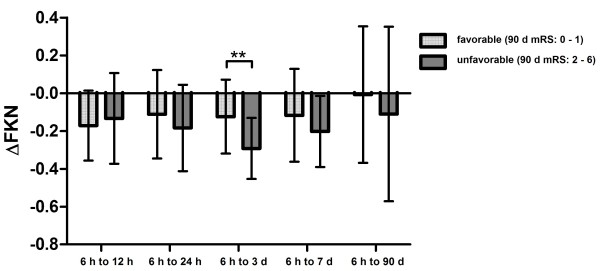
Proportional differences of fractalkine levels (∆FKN) at different time points after stroke depending on 90-day (d) stroke outcome (means ± SD).

Total levels of FKN were not different between patients with favorable (mRS: 0 to 1) and unfavorable 7-d or 90-d outcome (mRS: 2 to 6) at any time point (*P*>0.05). Since significant alterations were found between the initial levels and follow-up time points regarding stroke severity, we compared ∆FKN between patients with favorable and unfavorable outcome. Patients with favorable outcome showed significantly less decrease of FKN than patients with unfavorable outcome. There were differences of ∆FKN for the time interval 6 h to 3 d (*P* = 0.011), as well as 6 h to 90 d (*P* = 0.009) regarding the 7-d outcome (Figure 2). Also, we found differences of ∆FKN between 6 h and 3 d (*P* = 0.002) regarding the 90-d outcome (Figure 3). In a multivariate analysis including stroke severity, gender and stroke etiology according to TOAST criteria, it was analyzed whether ∆FKN was independently associated with unfavorable 90-d outcome. The logistic regression analysis revealed an independent association of ∆FKN between 6 h and 3 d with 90-d outcome (*P* = 0.031, OR = 0.011).

### Correlation of fractalkine with brain damage and inflammatory markers and leucocyte counts

In the control group, FKN levels correlated positively with CRP and TIMP-1 (Table 2). In stroke patients ∆FKN between 6 h and 3 d inversely correlated with S100B at 24 h (r = -0.441, *P* = 0.001) [see Additional file 1: Table S7]. There was no correlation between FKN levels or ∆FKN with inflammatory markers or leucocyte counts in the stroke patient group considering correction for multiple comparisons [see Additional file 1: Table S3-S6 and Additional file 1: Table S8].

**Table 2:** Correlations of fractalkine to brain damage and inflammation markers within the control cohort

	**R**	***P***
FKN versus CRP	0.562	0.001^a^
FKN versus MCP-1	0.391	0.027
FKN versus TIMP-1	0.676	<0.001^a^
FKN versus MMP-9	0.402	0.023
FKN versus IL-6	0.227	0.211
FKN versus S100B	-0.105	0.566

^a^*P*<0.008: significant after Bonferroni correction.

## Discussion

The main findings of this study are: (i) FKN levels in patients with moderate to severe ischemic stroke differ from controls at distinct time points; (ii) after ischemic stroke, the temporal profile of FKN levels differs in regard to stroke severity; (iii) dynamics of FKN are independently associated with neurological outcome; (iv) dynamics of FKN inversely correlate with the extent of brain damage as measured by S100B; and (v) the correlation between FKN and CRP and TIMP-1 that is present in healthy controls is not present in stroke patients, neither in the acute nor in the prolonged stage.

In this study, we observed an alteration of FKN levels after stroke compared to controls with CVRF, depending on stroke severity. Although we provide early levels of fractalkine, we cannot give any information about FKN dynamics in the phase before 6 h after stroke. However, at 3 d, at 7 d, and by tendency, at 90 d in patients with moderate/severe strokes, FKN levels are reduced compared to controls, while levels in patients with mild strokes are not. With this study, we are not able to answer whether FKN is released into plasma reactively, or whether it is itself intervening in the cascade of post-ischemic inflammation. At 90 days after stroke - a time point, at which post-stroke inflammation has decreased - FKN levels still differ between patients with mild and moderate/severe strokes. This might hint at an individual potential for regulating FKN. Furthermore, FKN might be rapidly regulated after the onset of stroke, since it differs at early time points after stroke depending on stroke severity.

Donohue *et al*. described the levels of FKN being linked to long-term outcome as measured by 180d-mRS [14]. Since the cascade of inflammation after acute ischemic stroke evolves rapidly, we aimed to investigate the association between early FKN levels and clinical outcome. Regarding the absolute levels of FKN, we could not find an association with outcome but could demonstrate a linkage only to stroke severity on admission. However, in the current study we could demonstrate that less decrease of FKN over time is related to a beneficial stroke outcome. In concordance to that, less decrease of FKN is also associated to smaller infarction sizes, represented by lower values of the damage marker S100B. This might support the assumption of an important linkage of FKN with the pathology of stroke.

Although there is some controversy about its distinct functions, the chemokine FKN has been regarded to fulfill a neuroprotective role in several *in vitro* and *in vivo* models of neurodegenerative and neuroinflammatory diseases. One hypothesis is that the interaction of the neuron-expressed FKN and its corresponding receptor, CX3CR1, which is found on microglia, leads to an inhibition of microglial destructive inflammation [4,5,20,21]. Treatment of microglia with FKN *in vitro*, for example, provokes an inhibition of cell death mechanisms through Fas ligand-induced cell death and other pro-apoptotic mechanisms [22]. Furthermore, it has been shown in a cell culture model that survival of hippocampal neurons can be induced by the presence of FKN via the protein kinase Akt pathway [23]. High levels of FKN might also play a role in cell death after stroke.

The main differences in ∆FKN were observed for the time interval from 6 h to 3 d. This could be because FKN may take part in the recruitment of leucocytes. In a murine stroke model, it has been shown that the influx of immune cells (for example, macrophages, lymphocytes and neutrophils) peaks at 1 to 3 d after ischemia [24]. Additionally, the infiltration of macrophages in the post-ischemic brain significantly increases between day 1 and day 4 after an experimental stroke [25]. Especially the nonclassical subset of monocytes, which expresses high levels of CX3CR1, could be of particular importance in the FKN-dependent recruitment of cells. Urra *et al*. reported a significant proportional decrease of nonclassical monocytes at 2 d after stroke onset. They also described an inverse association of these cells with infarct size and outcome [26]. This might be in line with our findings about the inverse association of ∆FKN with infarct size and outcome. Although it has been shown that monocyte subsets are involved in the post-ischemic inflammatory processes [27], detailed investigations of monocyte recruitment in connection with FKN and other chemokines to the brain are lacking. In a murine model of myocardial infarction, Ly-6C^low^ monocytes, which are comparable to the human nonclassical subset, dominate a second phase of inflammatory response, which starts at some days after ischemia and have been regarded as reparative [28] (for review see: [29]). Recruitment of Ly-6C^low^ monocytes to the post-ischemic myocardium is executed via FKN. Also after stroke, the migration of potentially protective nonclassical monocytes might be dependent on FKN. Possibly, a neuroprotective characteristic of FKN could, therefore, also be explained by a chemotactic impact on nonclassical monocytes. Measurements of the expression of CX3CR1 on circulating leukocytes would be helpful in future studies to address this point and to investigate the association of receptor expression and plasma FKN dynamics. While the experimental data suggest a potential neuroprotective effect of FKN, its impact in patients is uncertain. So far, it even remains unclear if concentrations of FKN in plasma are accurately reflecting its actions in the central nervous system.

FKN is also present on the surface of endothelial cells and contributes to chemotaxis and invasion of inflammatory cells, such as, for example, monocytes [30,31]. The CX3CL1/CX3CR1 axis is therefore suspected to carry out pro-atherogenetic functions, as it might contribute to formation of atherosclerotic lesions [32-35]. In our control collective, which contains individuals with cardiovascular risk factors, CRP levels are strongly correlated to FKN, implying that there might be a linkage to cardiovascular risk in humans. Recently, FKN was suggested to be an independent predictor of cardiovascular mortality in a heart failure patient cohort [36]. As FKN levels might be distorted by atherosclerotic diseases we also included the etiology of stroke in our multivariate model. But, ∆FKN was independently associated with clinical stroke outcome.

While FKN levels correlate significantly with CRP and TIMP-1 in healthy controls, there is no association in stroke patients. The fact that the associations are not restored until 90 d after stroke may underline the long-term impact of the acute event. The finding that there are no correlations to other markers of inflammation in stroke patients is in accordance to the different specific dynamics of FKN in stroke.

Although we provide evidence of an independent association of FKN dynamics with stroke outcome, and therefore present FKN as a putative future biomarker in stroke, the interpretation of the data must be done cautiously. Considering the multifactorial pathology of ischemic stroke, the patient cohort is relatively small. For that reason, we also could not evaluate a probable effect of any treatment, such as, for example, thrombolysis. Furthermore, despite strict exclusion criteria, an overlap of pre-ischemic inflammation processes that might distort the results cannot be ruled out completely. Due to strict exclusion criteria concerning infectious diseases, we could include relatively fewer patients with severe strokes. Finally, as this is a retrospective study, measurements of CX3CR1 expression on vital leucocytes could not be done.

## Conclusions

In this study, we investigated, for the first time, the behavior of FKN levels in the hyperacute stage after ischemic stroke in humans. Early FKN dynamics are independently related to stroke outcome. Supported by former animal and *in vitro* studies, FKN might be involved in the inflammatory cascade after acute ischemic stroke and could serve as a new biomarker, especially concerning its rapid regulation. However, due to the nature of the study we only can provide associations of FKN with stroke but no causality. The exact mechanisms need to be illuminated in future studies.

## Abbreviations

ADAM10: a disintegrin and metalloproteinase domain-containing protein 10; CRP: C-reactive protein; CVRF: cardiovascular risk factors; FKN: fractalkine; IL-6: interleukin 6; MCP-1: monocyte chemotactic protein 1; MMP-9: Matrix metallopeptidase 9; mRS: modified Rankin Scale; NIHSS: National Institutes of Health Stroke Scale; pMCAO: permanent occlusion of the middle cerebral artery; rtPA: reverse tissue-type plasminogen activator; TIMP-1: tissue inhibitors of metalloproteinases 1.

## Competing interests

The authors declare that they have no competing interests.

## Authors’ contributions

HW contributed to the conception and design of the study, acquisition of data, analysis, interpretation of data, drafting and revising the manuscript. GMG contributed to the conception and design of the study, analysis, interpretation of data, drafting and revising the manuscript. KW contributed to the conception and design of the study, acquisition of data, analysis, interpretation of data and revising the manuscript. ABT, MD, RS and HP contributed to acquisition of data, analysis, interpretation of data and revising the manuscript. RL contributed to the acquisition of data and revising the manuscript. All authors read and approved the final manuscript.

## Supplementary Material

Additional file 1: Table S3 Association of fractalkine with markers of inflammation within the stroke patient group. **Table S4** Association of fractalkine with S100B within the stroke patient group. **Table S5** Association of fractalkine with blood cell counts within the stroke patient group. **Table S6** Association of proportional differences of fractalkine (∆FKN) with markers of inflammation within the stroke patient group. **Table S7** Association of proportional differences of fractalkine (∆FKN) with S100B within the stroke patient group. **Table S8** Association of proportional differences of fractalkine (∆FKN) with blood cell counts within the stroke patient group. Click here for file
