# Supplementary material for: The temporal dynamics of plasma fractalkine levels in ischemic stroke: association with clinical severity and outcome
Source: J Neuroinflammation. 2014 Apr 10;11:74. doi: 10.1186/1742-2094-11-74 (PMC4022085; doi:10.1186/1742-2094-11-74)
Supplement: Additional file 1: Table S3 — Association of fractalkine with markers of inflammation within the stroke patient group. Table S4 Association of fractalkine with S100B within the stroke patient group. Table S5 Association of fractalkine with blood cell counts within the stroke patient group. Table S6 Association of proportional differences of fractalkine (∆FKN) with markers of inflammation within the stroke patient group. Table S7 Association of proportional differences of fractalkine (∆FKN) with S100B within the stroke patient group. Table S8 Association of proportional differences of fractalkine (∆FKN) with blood cell counts within the stroke patient group. [file 1742-2094-11-74-S1.docx]

**Additional files**

**Table S3.** Association of Fractalkine with markers of inflammation within the stroke patient group.

|  | | 6h | 12h | 24h | 3d | 7d | 90d |
| --- | --- | --- | --- | --- | --- | --- | --- |
| FKN vs. CRP | r | .046 | -.069 | -.101 | -.087 | -.100 | -.129 |
|  | p | .737 | .619 | .465 | .534 | .468 | .381 |
|  | n | 55 | 54 | 54 | 53 | 55 | 48 |
| FKN vs. MCP-1 | r | .173 | .307 | .281 | .137 | .134 | .247 |
|  | p | .206 | .022 | .037 | .322 | .331 | .088 |
|  | n | 55 | 55 | 55 | 54 | 55 | 49 |
| FKN vs. TIMP-1 | r | .113 | .119 | -.012 | -.184 | .040 | -.020 |
|  | p | .426 | .401 | .931 | .191 | .778 | .892 |
|  | n | 52 | 52 | 52 | 52 | 52 | 47 |
| FKN vs. MMP-9 | r | -.146 | -.187 | -.102 | -.139 | -.053 | .366 |
|  | p | .293 | .180 | .462 | .320 | .705 | .009 |
|  | n | 54 | 53 | 54 | 53 | 54 | 50 |
| FKN vs. IL-6 | r | -.041 | -.001 | -.131 | -.134 | .101 | .143 |
|  | p | .765 | .992 | .351 | .340 | .461 | .332 |
|  | n | 55 | 54 | 53 | 53 | 55 | 48 |

* p < 0.002: significant after Bonferroni correction.

**Table S4.** Association of Fractalkine with S100B within the stroke patient group.

|  |  | 6h | 12h | 24h | 3d | 7d | 90d |
| --- | --- | --- | --- | --- | --- | --- | --- |
| FKN vs. S100B | r | .117 | .055 | .040 | -.099 | .041 | -.099 |
|  | p | .393 | .692 | .774 | .482 | .766 | .508 |
|  | n | 55 | 54 | 54 | 53 | 55 | 47 |

* p < 0.05: significant.

**Table S5.** Association of Fractalkine with blood cell counts within the stroke patient group.

|  |  | 6h | 12h | 24h | 3d | 7d | 90d |
| --- | --- | --- | --- | --- | --- | --- | --- |
| FKN vs.  Leucocyte-Count | r | -.080 | -.064 | -.289 | -.303 | -.120 | -.130 |
|  | p | .562 | .641 | .032 | .024 | .382 | .369 |
|  | n | 55 | 55 | 55 | 55 | 55 | 50 |
| FKN vs.  Hemoglobine | r | -.048 | -.015 | -.056 | .004 | -.071 | .058 |
|  | p | .726 | .912 | .682 | .979 | .607 | .687 |
|  | n | 55 | 55 | 55 | 55 | 55 | 50 |
| FKN vs.  Hematocrit | r | -.045 | -.041 | -.045 | -.014 | -.091 | .030 |
|  | p | .743 | .764 | .746 | .918 | .507 | .835 |
|  | n | 55 | 55 | 55 | 55 | 55 | 50 |
| FKN vs.  Thrombocyte-Count | r | -.202 | -.293 | -.350 | -.384 | -.328 | -.162 |
|  | p | .138 | .030 | .009 | .004 | .015 | .260 |
|  | n | 55 | 55 | 55 | 55 | 55 | 50 |
| FKN vs.  Neutrophil-Count | r | .011 | .084 | -.175 | -.190 | -.020 | -.157 |
|  | p | .935 | .547 | .205 | .170 | .884 | .282 |
|  | n | 54 | 54 | 54 | 54 | 54 | 49 |
| FKN vs.  Lymphocyte-Count | r | -.228 | -.257 | -.186 | -.215 | -.210 | -.043 |
|  | p | .097 | .060 | .178 | .118 | .128 | .771 |
|  | n | 54 | 54 | 54 | 54 | 54 | 49 |
| FKN vs.  Monocyte-Count | r | -.085 | -.110 | -.323 | -.207 | -.200 | -.293 |
|  | p | .539 | .428 | .017 | .134 | .148 | .041 |
|  | n | 54 | 54 | 54 | 54 | 54 | 49 |
| FKN vs.  Eosinophil-Count | r | -.160 | -.092 | -.081 | -.022 | -.115 | .098 |
|  | p | .247 | .510 | .559 | .875 | .409 | .504 |
|  | n | 54 | 54 | 54 | 54 | 54 | 49 |
| FKN vs.  Basophil-Count | r | -.060 | -.079 | -.058 | -.033 | -.209 | -.055 |
|  | p | .666 | .569 | .677 | .811 | .130 | .707 |
|  | n | 54 | 54 | 54 | 54 | 54 | 49 |

* p < 0.001: significant after Bonferroni correction.

**Table S6.** Association of proportional differences of Fractalkine (∆FKN) with markers of inflammation within the stroke patient group.

|  | | ∆FKN  6h-12h | ∆FKN  6h-24h | ∆FKN  6h-3d | ∆FKN  6h-7d | ∆FKN  6h-90d |
| --- | --- | --- | --- | --- | --- | --- |
| CRP 6h | r | -.118 | -.160 | -.117 | -.101 | .134 |
|  | p | .391 | .243 | .395 | .461 | .354 |
|  | n | 55 | 55 | 55 | 55 | 50 |
| CRP 12h | r | -.104 | -.166 | -.130 | -.110 | .099 |
|  | p | .453 | .229 | .349 | .429 | .498 |
|  | n | 54 | 54 | 54 | 54 | 49 |
| CRP 24h | r | -.084 | -.208 | -.194 | -.164 | -.131 |
|  | p | .547 | .132 | .160 | .237 | .370 |
|  | n | 54 | 54 | 54 | 54 | 49 |
| CRP 3d | r | -.063 | -.122 | -.148 | -.130 | -.184 |
|  | p | .656 | .384 | .290 | .354 | .205 |
|  | n | 53 | 53 | 53 | 53 | 49 |
| CRP 7d | r | .122 | -.126 | -.175 | -.098 | -.090 |
|  | p | .374 | .358 | .201 | .475 | .535 |
|  | n | 55 | 55 | 55 | 55 | 50 |
| CRP 90d | r | .237 | .087 | .089 | -.003 | -.196 |
|  | p | .101 | .553 | .544 | .983 | .182 |
|  | n | 49 | 49 | 49 | 49 | 48 |
| MCP-1 6h | r | .187 | .163 | -.131 | .003 | -.053 |
|  | p | .172 | .234 | .339 | .981 | .716 |
|  | n | 55 | 55 | 55 | 55 | 50 |
| MCP-1 12h | r | .099 | .080 | .023 | .041 | -.063 |
|  | p | .471 | .562 | .867 | .767 | .664 |
|  | n | 55 | 55 | 55 | 55 | 50 |
| MCP-1 24h | r | .177 | -.007 | -.027 | -.087 | -.239 |
|  | p | .196 | .959 | .843 | .527 | .094 |
|  | n | 55 | 55 | 55 | 55 | 50 |
| MCP-1 3d | r | .189 | .112 | -.022 | .011 | -.089 |
|  | p | .171 | .420 | .872 | .937 | .539 |
|  | n | 54 | 54 | 54 | 54 | 50 |
| MCP-1 7d | r | .138 | .020 | -.073 | -.012 | -.094 |
|  | p | .315 | .883 | .595 | .934 | .517 |
|  | n | 55 | 55 | 55 | 55 | 50 |
| MCP-1 90d | r | .082 | .090 | .032 | .078 | -.081 |
|  | p | .573 | .540 | .826 | .594 | .579 |
|  | n | 49 | 49 | 49 | 49 | 49 |
| TIMP-1 6h | r | .108 | .021 | -.109 | -.104 | .008 |
|  | p | .445 | .884 | .442 | .461 | .958 |
|  | n | 52 | 52 | 52 | 52 | 48 |
| TIMP-1 12h | r | .050 | -.020 | -.066 | -.233 | -.161 |
|  | p | .726 | .889 | .640 | .096 | .276 |
|  | n | 52 | 52 | 52 | 52 | 48 |
| TIMP-1 24h | r | .048 | .005 | -.217 | -.229 | -.104 |
|  | p | .736 | .971 | .123 | .103 | .483 |
|  | n | 52 | 52 | 52 | 52 | 48 |
| TIMP-1 3d | r | .092 | -.065 | -.270 | -.215 | -.076 |
|  | p | .514 | .648 | .053 | .126 | .606 |
|  | n | 52 | 52 | 52 | 52 | 48 |
| TIMP-1 7d | r | .080 | -.047 | -.279 | -.163 | -.003 |
|  | p | .571 | .742 | .046 | .248 | .983 |
|  | n | 52 | 52 | 52 | 52 | 48 |
| TIMP-1 90d | r | .264 | .178 | -.101 | -.145 | -.007 |
|  | p | .073 | .231 | .499 | .330 | .965 |
|  | n | 47 | 47 | 47 | 47 | 47 |
| MMP-9 6h | r | -.089 | -.116 | -.115 | -.086 | .115 |
|  | p | .521 | .405 | .408 | .535 | .427 |
|  | n | 54 | 54 | 54 | 54 | 50 |
| MMP-9 12h | r | .031 | .015 | -.209 | .005 | .183 |
|  | p | .825 | .913 | .133 | .971 | .209 |
|  | n | 53 | 53 | 53 | 53 | 49 |
| MMP-9 24h | r | .006 | .021 | -.175 | -.084 | .165 |
|  | p | .967 | .877 | .205 | .547 | .253 |
|  | n | 54 | 54 | 54 | 54 | 50 |
| MMP-9 3d | r | -.094 | -.149 | -.162 | -.104 | .187 |
|  | p | .504 | .286 | .248 | .459 | .198 |
|  | n | 53 | 53 | 53 | 53 | 49 |
| MMP-9 7d | r | .016 | .037 | -.053 | .007 | .092 |
|  | p | .911 | .790 | .705 | .960 | .523 |
|  | n | 54 | 54 | 54 | 54 | 50 |
| MMP-9 90d | r | -.133 | .004 | .107 | -.013 | .225 |
|  | p | .356 | .979 | .458 | .926 | .116 |
|  | n | 50 | 50 | 50 | 50 | 50 |
| IL6 6h | r | .159 | .089 | -.326 | -.096 | -.077 |
|  | p | .246 | .518 | .015 | .484 | .595 |
|  | n | 55 | 55 | 55 | 55 | 50 |
| IL6 12h | r | -.057 | -.220 | -.241 | -.123 | .019 |
|  | p | .681 | .110 | .079 | .375 | .898 |
|  | n | 54 | 54 | 54 | 54 | 49 |
| IL6 24h | r | .028 | -.290 | -.404 | -.242 | -.241 |
|  | p | .844 | .035 | .003 | .081 | .099 |
|  | n | 53 | 53 | 53 | 53 | 48 |
| IL6 3d | r | -.043 | -.153 | -.186 | -.107 | -.005 |
|  | p | .757 | .274 | .182 | .447 | .975 |
|  | n | 53 | 53 | 53 | 53 | 49 |
| IL6 7d | r | .102 | -.153 | -.286 | -.139 | -.135 |
|  | p | .460 | .266 | .034 | .313 | .350 |
|  | n | 55 | 55 | 55 | 55 | 50 |
| IL6 90d | r | .300^*^ | .117 | -.021 | -.147 | -.166 |
|  | p | .036 | .425 | .889 | .315 | .259 |
|  | n | 49 | 49 | 49 | 49 | 48 |

* p < 0.0003: significant after Bonferroni correction

**Table S7.** Association of proportional differences of Fractalkine (∆FKN) with S100B within the stroke patient group.

|  |  | ∆FKN  6h-12h | ∆FKN  6h-24h | ∆FKN  6h-3d | ∆FKN  6h-7d | ∆FKN  6h-90d |
| --- | --- | --- | --- | --- | --- | --- |
| S100B 6h | r | -.141 | -.243 | -.391 | -.335 | -.156 |
|  | p | .305 | .074 | .003 | .012 | .278 |
|  | n | 55 | 55 | 55 | 55 | 50 |
| S100B 12h | r | .068 | -.129 | -.223 | -.227 | -.123 |
|  | p | .627 | .353 | .104 | .098 | .401 |
|  | n | 54 | 54 | 54 | 54 | 49 |
| S100B 24h | r | .116 | -.233 | **-.441^*^** | -.303 | -.284 |
|  | p | .404 | .090 | **.001** | .026 | .048 |
|  | n | 54 | 54 | **54** | 54 | 49 |
| S100B 3d | r | -.061 | .041 | -.135 | -.064 | -.015 |
|  | p | .662 | .768 | .334 | .647 | .919 |
|  | n | 53 | 53 | 53 | 53 | 49 |
| S100B 7d | r | .031 | -.174 | -.261 | -.124 | -.170 |
|  | p | .823 | .205 | .054 | .368 | .238 |
|  | n | 55 | 55 | 55 | 55 | 50 |
| S100B 90d | r | .238 | .025 | -.105 | -.093 | -.175 |
|  | p | .103 | .866 | .476 | .529 | .239 |
|  | n | 48 | 48 | 48 | 48 | 47 |

* p < 0.002: significant after Bonferroni correction.

**Table S8.** Association of proportional differences of Fractalkine (∆FKN) with blood cell counts within the stroke patient group.

|  |  | ∆FKN  6h-12h | ∆FKN  6h-24h | ∆FKN  6h-3d | ∆FKN  6h-7d | ∆FKN  6h-90d |
| --- | --- | --- | --- | --- | --- | --- |
| Leucocyte-Count | r | .207 | -.083 | -.319 | .001 | .052 |
|  | p | .130 | .549 | .018 | .997 | .721 |
|  | n | 55 | 55 | 55 | 55 | 50 |
| Hemoglobine | r | -.025 | -.092 | -.083 | .028 | .147 |
|  | p | .855 | .504 | .549 | .837 | .310 |
|  | n | 55 | 55 | 55 | 55 | 50 |
| Hematocrit | r | -.054 | -.057 | -.086 | .008 | .123 |
|  | p | .696 | .681 | .533 | .952 | .394 |
|  | n | 55 | 55 | 55 | 55 | 50 |
| Thrombocyte-Count | r | -.009 | -.065 | -.202 | -.202 | -.013 |
|  | p | .948 | .637 | .139 | .139 | .930 |
|  | n | 55 | 55 | 55 | 55 | 50 |
| Neutrophil-Count | r | .188 | -.198 | -.359 | -.048 | -.113 |
|  | p | .173 | .151 | .008 | .731 | .440 |
|  | n | 54 | 54 | 54 | 54 | 49 |
| Lymphocyte-Count | r | .076 | .271 | .061 | .142 | .342 |
|  | p | .586 | .048 | .661 | .304 | .016 |
|  | n | 54 | 54 | 54 | 54 | 49 |
| Monocyte-Count | r | -.004 | -.320 | -.250 | -.059 | -.030 |
|  | p | .977 | .018 | .068 | .671 | .838 |
|  | n | 54 | 54 | 54 | 54 | 49 |
| Eosinophil-Count | r | .099 | .134 | .240 | .035 | .356 |
|  | p | .478 | .334 | .081 | .801 | .012 |
|  | n | 54 | 54 | 54 | 54 | 49 |
| Basophil-Count | r | -.153 | -.056 | -.055 | -.269 | -.108 |
|  | p | .270 | .686 | .694 | .049 | .460 |
|  | n | 54 | 54 | 54 | 54 | 49 |

* p < 0.001: significant after Bonferroni correction.
